# ﻿*Microdousamblyrhynchos* sp. nov., a new member of the small-toothed sleepers (Teleostei, Gobiiformes, Odontobutidae) from Guangxi, southern China

**DOI:** 10.3897/zookeys.1153.97139

**Published:** 2023-03-10

**Authors:** Jiantao Hu, Chun Lan, Chenhong Li

**Affiliations:** 1 Shanghai Universities Key Laboratory of Marine Animal Taxonomy and Evolution, Shanghai Ocean University, Shanghai 201306, China; 2 Engineering Research Center of Environmental DNA and Ecological Water Health Assessment, Shanghai Ocean University, Shanghai 201306, China; 3 Guangxi Duan Yao Autonomous County Aquatic Technology Station, Duan 530700, China

**Keywords:** Freshwater sleepers, morphology, phylogeny, taxonomy, Xijiang River

## Abstract

*Microdousamblyrhynchos*, a new species, the second one in the genus, from the family Odontobutidae, is described from the Hongshui River, in the upper reaches of the Xijiang River of the Pearl River drainage, Baise City, Guangxi Zhuang Autonomous Region, southern China. This species is distinguished from its only congener, *M.chalmersi*, by the blunt snout (vs. pointed); mean snout length/head length ratio 0.27 (vs. 0.3); eye not extending outward (vs. protruding); mean interorbital width/head length ratio 0.25 (vs. 0.11). Additionally, the results of molecular phylogenetic analysis confirmed that *M.amblyrhynchos***sp. nov.** is distinct from its sister species, *M.chalmersi*.

## ﻿Introduction

The family Odontobutidae comprises about 15–22 species in seven genera (Iwata 2011; Li et al. 2018). They are typically small, benthic, ambush predators that are endemic to freshwater ponds and creeks of eastern and southern Asia. This family can be distinguished from other gobiiforms by the following combination of characters: (1) six branchiostegal rays; (2) separated pelvic fins; (3) well-developed scapula excluding the cleithrum and proximal radials; and (4) longitudinally distributed lines of cephalic sensory papillae (Hoese and Gill 1993; Iwata 2011; Li et al. 2018). Li et al. (2018) revealed two clades within Odontobutidae using nuclear gene capture data, one of which includes three monophyletic genera: *Microdous* Li, 2018 (southern China and northern Vietnam) as sister to a clade consisting of *Micropercops* Fowler & Bean, 1920 (China, Japan and the Korean Peninsula) and *Sineleotris* Herre, 1940 (southern China and the Indo-China Peninsula).

*Microdous* Li, He, Jang, Liu & Li, 2018 was established for *Phylipnuschalmersi* Nichols & Pope, 1927 and distinguished from other genera of Odontobutidae by a unique combination of the following character states: (1) presence of suborbital bone; (2) presence of complete cephalic sensory canals; (3) small and cuspidal gill rakers; (4) gill openings extending to under the front part of the eyes; (5) absence of vertical bands on the sides; (6) absence of a dark band under the eye; and (7) presence of an irregular black fleck on the upper part of the base of the pectoral fin in preserved specimens (Li et al. 2018). In the present study, a new species in the previously monotypic *Microdous* is described from Guangxi, southern China. The new species is also supported by a molecular phylogenetic analysis of mitochondrial cytochrome c oxidase subunit I (COI) implemented to evaluate the position of the new species among closely related taxa.

## ﻿Materials and methods

**Figure 1.**
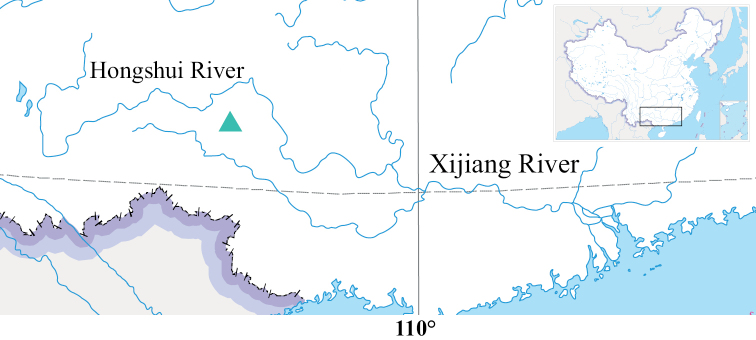
The sampling location of *Microdousamblyrhynchos* sp. nov. in China (inset) indicated by the green triangle.

In total, eight specimens were collected from Lihong Village (24°26.21'N, 106°26.72'E; c. 870 m a.s.l.), Yuhong Town, Lingyun County, Baise City, Guangxi Zhuang Autonomous Region using fish traps (Fig. 1). Specimens were preserved in 95% ethanol in the field then transferred to 75% ethanol. The right pectoral fins of six specimens were clipped for molecular analysis, and then the specimens were fixed in 10% formalin and transferred into 75% ethanol for morphological examination. All specimens were assigned with a collection number (CL) to facilitate sample tracking. Five specimens (holotype and four paratypes) were used to collect morphological measurements and most counts except for the number of gill rakers and vertebrae (Table 1). Three paratypes were used in the molecular analysis. Seven specimens were used to calculate snout length/ head length ratios in order to examine whether the snout was blunter in the new species than in that of *M.chalmersi*.

**Table 1.** Morphological characters for the type specimens of *Microdousamblyrhynchos* sp. nov.

Characters	SOU1801010-7	SOU1801010-8	SOU1801010-1	SOU1801010-2	SOU1801010-3
**Counts**					
Dorsal fins	VIII;I/9	VII;I/9	VII;I/9	VII;I/9	VIII;I/10
Pectoral fins	15	15	15	15	15
Pelvic fins	I/5	I/5	I/5	I/5	I/5
Anal fin	I/8	I/8	I/8	I/8	I/8
Caudal fin	15	15	15	15	15
Lateral scales	41	40	40	40	41
Transverse scales	13	13	13	13	13
Predorsal scales	24	23	21	21	23
Gill rakers	4+9 (counted on a non-type specimen)				
Vertebrae	16+18 (counted on a non-type specimen)				
**Measurements**					
Total length (TL), mm	89.9	81.6	83.6	75.4	90.2
Standard length (SL), mm	74.9	66.9	69.8	63.2	76.2
**Percentage of SL (%)**					
Head length	30.1	27.9	30.3	31.3	29.2
Predorsal length	35.8	36.1	37.4	39.4	37.2
Snout to second dorsal fin	54.8	55.1	57.6	55.7	54.9
Snout to anus	56.5	55.5	55.5	57.0	56.1
Snout to pelvic fins	31.0	30.3	31.2	32.1	29.5
Length of caudal peduncle	26.9	26.3	26.1	25.6	24.9
Depth of caudal peduncle	11.7	10.4	10.8	10.6	10.2
Body depth at first dorsal fin origin	19.0	17.0	19.4	18.6	18.2
Body width at anal fin origin	12.9	9.1	12.0	9.5	10.0
Length of first dorsal fin base	12.5	13.6	14.3	11.3	14.7
Length of second dorsal fin base	20.1	19.5	20.0	18.9	19.3
Length of anal fin base	12.6	13.1	15.3	12.8	13.0
Length of pectoral fin	22.0	20.6	22.6	23.8	22.0
Length of pelvic fin	15.9	17.7	18.4	19.9	18.5
**Percentage of head length (%)**					
Snout length	26.4	27.3	27.3	27.8	27.0
Maximum head depth	59.3	60.0	64.0	55.3	61.9
Maximum head width	61.0	62.7	64.9	58.0	62.2
Interorbital width	26.8	26.2	27.2	26.9	26.1
Eye diameter	21.0	21.6	19.2	20.4	22.1
Lower jaw length	31.6	36.5	33.3	28.4	33.4

Type specimens were deposited at the Fish Collection of the Shanghai Ocean University (**SOU**), China. Collection numbers and voucher numbers (in parentheses) of specimens of the new species are as follows: CL3084-1 (= SOU1801010-1), CL3084-2 (= SOU1801010-2), CL3084-3 (= SOU1801010-3), CL3084-4 (= SOU1801010-4), CL3084-5 (= SOU1801010-5), CL3084-6 (= SOU1801010-6), CL3084-7 (= SOU1801010-7), CL3084-8 (= SOU1801010-8).

Morphological measurements, counts and observations followed Wu et al. (2008). Nomenclature of cephalic sensory pores followed Akihito (1986). Each specimen was sexed by morphology of the urogenital papilla (Miller 1992). Two non-type specimens were dissected for counts of the number of gill rakers and vertebrae, and for examination of the vomerine region.

Genomic DNA was extracted using an Ezup Column Animal Genomic DNA Purification Kit (Sangon, Shanghai, China). Partial sequences (~ 1500 bp) of cytochrome c oxidase subunit I (COI), covering whole barcoding region was amplified from three paratype specimens (SOU1801010-1, SOU1801010-2, SOU1801010-3) as well as from comparative specimens (Table 2) using Vazyme 2× Taq Plus Master Mix II (Sangon, Shanghai) with a forward primer, CCATTTTACCTGTGRCAATCACACG, and a reverse primer CAGAGCGGTTATGTRTCTGGCTTGAA according to Zhou et al. (2022). The following thermal cycles was followed: an initial denaturation at 94 °C for 5 min, 30 cycles of denaturation at 94 °C for 30 sec, primer annealing at 55 °C for 30 sec, extension at 72 °C for 1 min, and the final extension was 7 min at 72 °C (Zhou et al. 2022). The amplified products were checked on 1% agarose gels before sending to Azenta (Suzhou, Jiangsu, China) for sequencing. The sequence of three paratype specimens were lodged in GenBank with accession numbers OP536373, OP536374 and OP536375.

**Table 2.** Localities, voucher information and GenBank accession numbers for the samples used in this study.

Species	Collection number	Locality	GenBank numbers
*Microdousamblyrhynchos*	SOU1801010-1 SOU1801010-2 SOU1801010-3	Baise, Guangxi, China	OP536373, OP536374, OP536375
*Microdouschalmersi*	CL2076-3	Wuzhishan, Hainan, China	OQ319987,
	CL2076-5		OQ319988
*Sineleotrissaccharae*	25012	Fangchenggang, Guangxi, China	OQ382855
*Neodontobutishainanensis*	20272	Hainan, China	OQ330750
*N.lani*	25911	Longzhou, Guangxi, China	OQ330749
*Micropercopsswinhonis*	–	–	NC_021763.1
*Odontobutissinensis*	–	–	NC_022818.1
*O.haifengensis*	–	–	NC_036056.1
*O.interruptus*	–	–	NC_027583.1
*O.potamophilus*	–	–	NC_022706.1
*O.platycephala*	–	–	NC_010199.1
*O.yaluensis*	–	–	NC_027160.1
*Perccottusglenii*	–	–	NC_020350.1
*Rhyacichthysaspro*	–	–	NC_004414.1

Sequences of additional material were retrieved from the National Center for Biotechnology Information database (Table 2). All sequences were aligned and analyzed using MEGA11 (Tamura et al. 2021). A maximum-likelihood tree including all genera of Odontobutidae except for *Terateleotris* was reconstructed under the HKY+G model with 1000 bootstrap replications. The resulting tree was visualized in FigTree v. 1.4.4 (Rambaut 2018).

## ﻿Taxonomic account

### ﻿ Microdous amblyrhynchos sp. nov.

Taxon classificationAnimaliaGobiiformesOdontobutidae

7CDE78D9-F945-543B-A13D-FE496C162965

https://zoobank.org/2C35228B-6C4A-4D19-B8EC-C0E03CB2D67E

**Figure 2.**
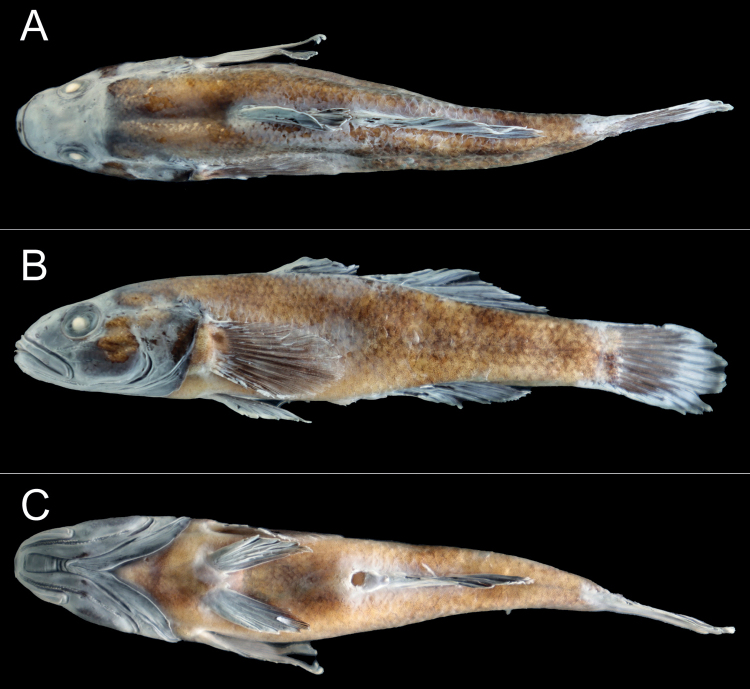
*Microdousamblyrhynchos* sp. nov., SOU1801010-7, holotype, 74.9 mm standard length, Baise City, Guangxi **A** dorsal **B** lateral **C** ventral.

**Figure 3.**
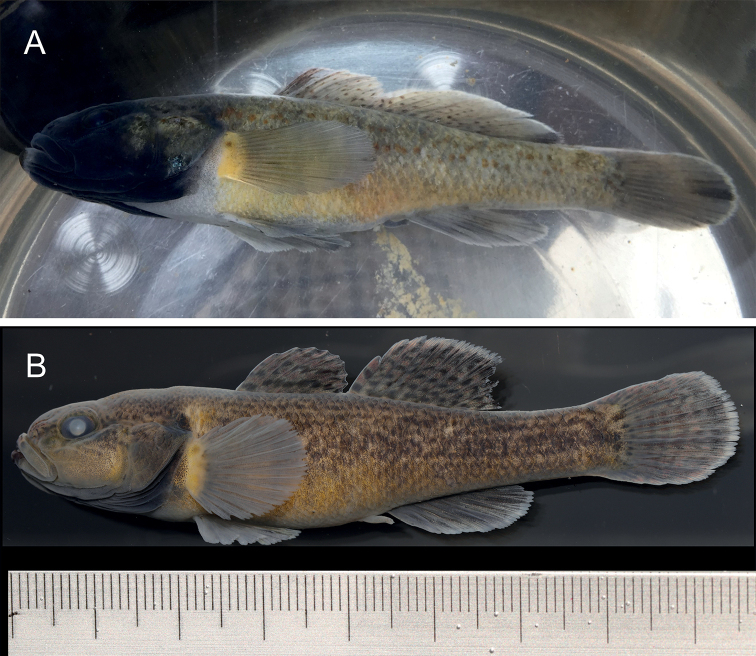
*Microdousamblyrhynchos* sp. nov. **A** live male individual with spawning coloration from a local market in Yuhong Town, Lingyun County, Baise City, Guangxi, China; photo taken in summer, 2019 **B** fresh non-type specimen, male, deposited at collection of Duan Yao Autonomous County Aquatic Technology Station, Guangxi; preserved in 10% formalin.

Figs 2 , 3 Blunt-snout small-toothed sleeper, Dùn Wěn Xì Chǐ Yoù, 钝吻细齿䱂 (Chinese)

#### Type material.

***Holotype*.** SOU1801010-7 (CL3084-7) (Fig. 2), male, 74.9 mm standard length (SL), obtained from an unnamed stream of a tributary of the Hongshui River, upper reaches of the Xijiang River of the Pearl River basin; Lihong Village (24°26.21'N, 106°26.72'E; c. 870 m a.s.l.), Yuhong Town, Lingyun County, Baise City, Guangxi, China (Fig. 1); collected using fish trap by J.H. Lan, May 2020.

***Paratypes*.** SOU1801010-8 (CL3084-8), female, 66.9 mm SL; SOU1801010-1 (CL3084-1), male, 69.8 mm SL; SOU1801010-2(CL3084-2), male, 63.2 mm standard length; SOU1801010-3 (CL3084-3), male, 76.2 mm SL. Sampling data same as for the holotype.

#### Diagnosis.

**Figure 4.**
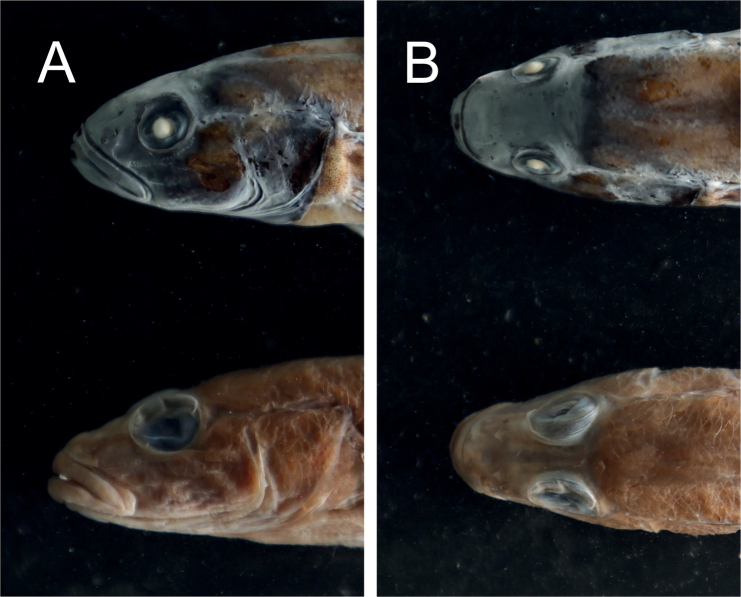
Head lateral (**A**) and dorsal (**B**) view of *Microdousamblyrhynchos* sp. nov. (upper, SL 74.9 mm, SOU1801010-7) and *M.chalmersi* (lower, SL 83.0 mm, SOU070504).

*Microdousamblyrhynchos* sp. nov. can be distinguished from the only congener *M.chalmersi* by the following character states: snout blunt, snout length/head length ratio 0.26–0.28, mean 0.27 (vs. pointed, snout length/head length ratio 0.28–0.32); eye large, but not protruding outward, interorbital width larger than eye diameter, interorbital width/head length ratio 0.22–0.27,mean = 0.25 (vs. large and protruding eye, interorbital width equal to or smaller than eye diameter, interorbital width/head length ratio 0.11–0.12) (Fig. 4).

#### Description.

Morphometric and meristic data for the holotype and paratypes are presented in Table 1. The maximum standard length (SL) was 76.2 mm.

First dorsal fin rays VII or VIII; second dorsal fin rays I/9–10; anal fin rays I/8–9; pectoral fin rays 15; pelvic fin rays I/5; caudal fin rays 15; longitudinal scale rows 40–41; transverse scale rows 13; predorsal scales 21–24; gill rakers 4+9; vertebrae 34 (16+18).

**Figure 5.**
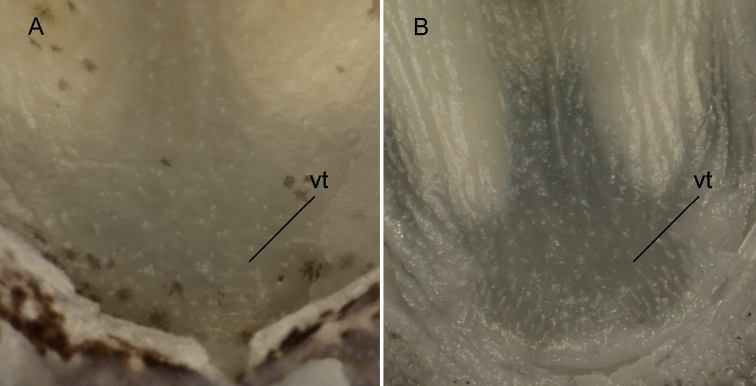
Vomer region **A***Microdousamblyrhynchos* sp. nov. SOU1801010-5 **B***M.chalmersi* CL2076-1. Abbreviation: vt, vomerine teeth-like dermal projections.

**Figure 6.**
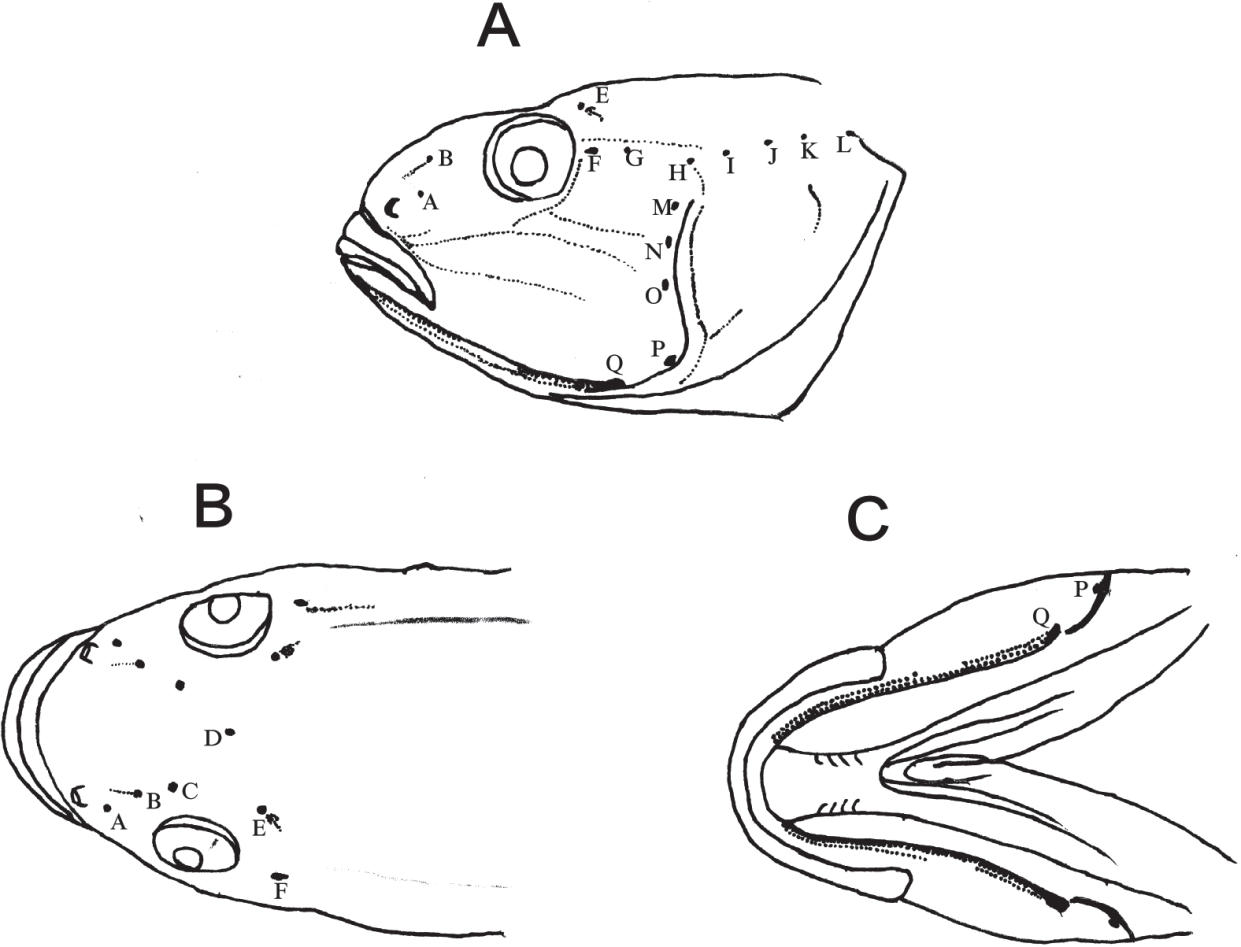
Patterns of main cephalic sensory papilla lines (rows of dots) and cephalic sensory canal system (red spots, A–Q) of *Microdousamblyrhynchos* sp. nov. (SOU1801010-7, holotype) in lateral (**A**), dorsal (**B**) and ventral (**C**) view.

**Figure 7.**
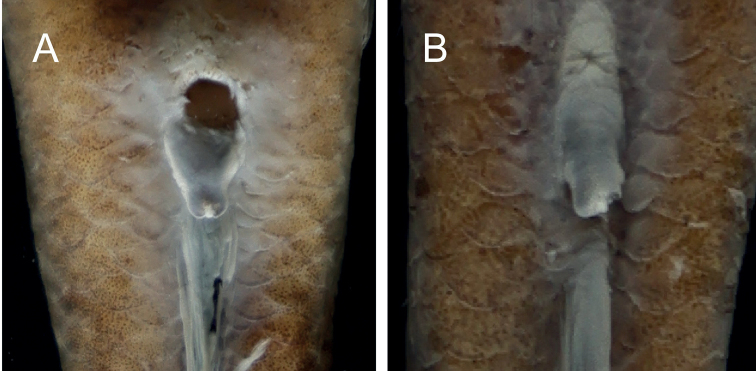
Urogenital papilla of *Microdousamblyrhynchos* sp. nov. SOU1801010-7 (**A** male) and SOU1801010-8 (**B** female).

Body stout, slightly compressed posteriorly. Head large, slightly depressed. Eye large, located in anterior half of head, not protruding outward. Several rows of tiny conical teeth on both jaws. Tiny, slender, teeth-like dermal projections in vomerine region (Fig. 5A). Gill opening extending to under anterior part of eye. Cephalic sensory canals complete (Fig. 6). Urogenital papilla distinct, rest behind the anus opening (Fig. 7).

Posterior tip of dorsal fin reaching origin of second dorsal fin when depressed. Second dorsal fin ends distinctly anterior of origin of caudal fin. Caudal fin and pectoral fin large, elliptical. Pelvic fins well separated, rear tip not reaching anus. Interopercle and subopercle naked. Ctenoid scales on dorsal and lateral side of body, ventral side of body posterior to anus and opercle, transforming ctenii present. Small cycloid scales on predorsal area, cheek, nape, preopercle, base of the pectoral fin, breast and abdomen.

***Coloration in life*.** Head black and dark brown with black dots on cheek. Body side yellowish, several irregular dark patches and orange dots on the side. Back dark brown. Unpaired fins possessing several inconspicuous stripes of dark spots and white edge. Pectoral fins and pelvic fins transparent and dusky. An irregular black fleck on upper part of base of pectoral fin. Ventral side of abdomen pale, with dull and inconspicuous dark patches. Urogenital papilla dark brown (Fig. 3A).

***Coloration preserved*.** Head dark brown with black dots on the cheek. Body side brown, orange dots absent. Back brown. Irregular black fleck on upper part of base of pectoral fin dull and inconspicuous. Ventral side of abdomen light brown. Urogenital papilla whitish (Fig. 3B).

***Sexual dimorphism*.** Urogenital papilla elongate with a wide base, tapering and with a narrow tip in male; truncated in female (Fig. 7).

***Cephalic sensory canals system*.** Anterior extension in front of interorbital with three pairs of pores A, B and C, and a single interorbital pore D. A pair of pores E lateral to pore D. Lateral section of oculoscapular canal with a series of seven pairs of pores: F to L (terminal). Preopercular canal extending to ventral side of preopercle, with five pairs of pores: M (dorsal) to Q (ventral) (Fig. 6).

***Cephalic sensory papillae*.** Neuromast numerous, small and densely set in mostly longitudinally arranged rows (Fig. 6).

#### Biology.

*Mircodousamblyrhynchos* can be found in small creeks or rivers with slow moving, clear water and rocky bottom. Some remains of chitin exoskeleton of crustaceans were found in the anus opening of the holotype, suggesting that *M.amblyrhynchos* is carnivorous. The male’s head would turn black with several orange dots on the body sides during their spawning seasons.

#### Etymology.

This species is named for its blunt snout distinguishing it from *M.chalmersi*. The species name derives from Greek *ambly* meaning dull or blunt and *rhynchos* meaning snout.

#### Phylogenetic analysis.

**Figure 8.**
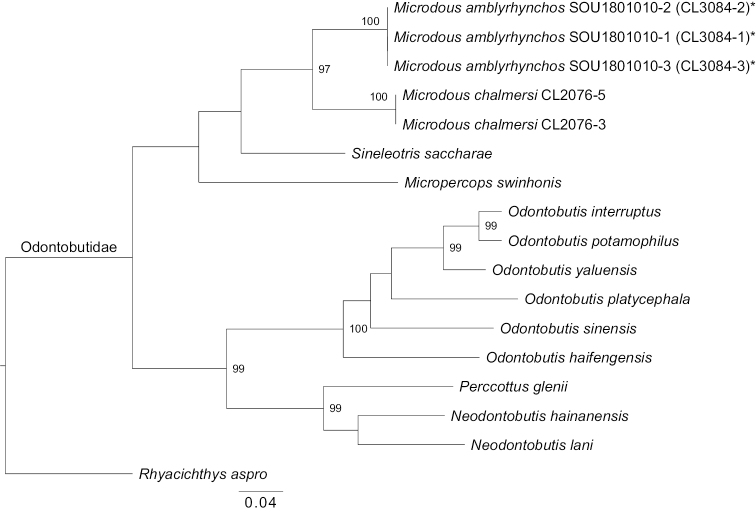
Maximum-likelihood (ML) tree of *Microdousamblyrhynchos* sp. nov., and other species of the Odontobutidae, based on sequences (~ 1500 bp) covering the whole barcoding region of cytochrome c oxidase subunit I (COI) gene. Numbers at nodes indicate the bootstrap support (BS). BS < 95 are not shown. SOU1801010-1, SOU1801010-2, and SOU1801010-3 are the voucher numbers of the paratypes. CL2076-3 and CL2076-5 are the collection numbers of *M.chalmersi*.

The COI tree with three individuals of *M.amblyrhynchos* and representative species of all available Odontobutidae genera except for *Terateleotris* is shown in Fig. 8. *Rhyacichthysaspro* Valenciennes, 1837 was used as the outgroup following Li et al. (2018). All genera of Odontobutidae were monophyletic in the tree. All *M.amblyrhynchos* specimens formed a clade distinct from *M.chalmersi*. The p-distance between *M.amblyrhynchos* and *M.chalmersi* was 10.7% (Suppl. material 1), suggesting that *M.amblyrhynchos* is a distinct species of the genus *Microdous*.

## ﻿Discussion

*Microdous* differs from other genera of the Odontobutidae by the following characters: lateral line absent (present in *Terateleotris*); barbel-like projection on sensory papilla absent (present in *Neodontobutis*); prevomerine teeth absent (present in *Perccottus*); cephalic sensory canals complete (moderate in *Micropercops*, absent or reduced in *Odontobutis*); maximum head width/maximum head depth ratio slightly greater or equal to 1 (vs. maximum head depth/maximum head width far less than 1 in *Sineleotris*); and dark band under eye (vs. present in *Sineleotris*).

Our analyses using both morphological and molecular data clearly suggest that the specimens collected from Yuhong Town, Lingyun County, Baise City, Guangxi, southern China should be recognized as a new species belonging to the genus *Microdous*. This new species can be morphologically distinguished from its only congener by its wider interorbital width, blunt snout, and non-extending eyes. A single specimen (as *Perccottuschalmersi*) from Xiajia village, Lingyun town was described by Zheng (1981) that had a blunt snout and dark fins with several inconspicuous stripes formed of dark spots. This specimen might in fact be a record of *M.amblyrhynchos*; however, it was inaccessible for comparison due to its unknown whereabouts.

The genus *Microdous* was established by Li et al. (2018) for *P.chalmersi* and the etymology of the generic name is “small + teeth” in Greek, referring to “a curved band of fine teeth on the vomer” (Nichols and Pope 1927) or “slender and tiny teeth on the vomer region of the fish” (Li et al. 2018), which were also mentioned as “vomerine teeth” in other studies (Zheng 1981; Wu et al. 2008). This character state is present in both *M.amblyrhynchos* and *M.chalmersi* (Fig. 5). However, our stereomicroscopic observation of the vomerine region showed that these projections growing directly from skin are different from the prevomerine teeth in other species such as *Perccottusglenii* Dybowski, 1877. Similar “skin teeth” were also found in *Neodontobutishainanensis* Chen, 2002 and *Sineleotrissaccharae* Herre, 1940 (Chen and Zheng 1985; Pan 1991). These vomerine teeth-like dermal projections are weak and tiny compared to the size of the fish, leaving their function as unclear.

Our phylogenetic analysis (Fig. 8) showed that *Microdous* is monophyletic, and the specimens of *M.amblyrhynchos* formed a clade distinct from *M.chalmersi*. *Microdous* was dated to the Miocene (17.9 Ma) and its origin was estimated as from southern China (Li et al. 2018). Biogeographical (Li 1981; Zhu 2016), palaeobotanical (Yao et al. 2009) and geological (Liang 2018) studies indicated that the Hainan Island was adjacent to the mainland several times due to plate movement, volcanic activity or sea level change since the Eocene epoch (30 Ma) (Liang 2018). Thus, the repeated connections and separations between Hainan Island and mainland could account for the divergence between *M.chalmersi* and *M.amblyrhynchos*.

The present-day distribution of genus *Microdous* includes southern China and northern Vietnam (Kottelat 2001 as *Perccottuschalmersi*). However, the Laotian species *Sineleotrisnamxamensis* (Chen & Kottelat, 2004) shares closest morphological similarities with the genus *Microdous* rather than the genus *Sineleotris*, such as the absence of a dark band under the eye and the arrangement of the cephalic sensory canal system. Thus, *S.namxamensis* would be better placed in the genus *Microdous*; the taxonomy of this species requires further research and molecular evidence.

Due to the rarity of the species, we know nothing about the reproduction, life cycle, and behavior of *M.amblyrhynchos*. Currently, the new species is only known from its type locality despite frequent surveys in Guangxi, suggesting that it is probably a species with a restricted distribution range. Habitat degradation at the type locality due to invasive species and illegal electric fishing may threaten the survival of this species. Therefore, we recommend that *M.amblyrhynchos* should be listed as a Vulnerable (VU) species [IUCN Red List criteria A1cde+B1b(iii)+D2].

According to Li et al. (2018), southern China maybe the region where the family Odontobutidae originated. This region has high Odontobutidae species diversity, including eight species of five genera. A new member of the genus *Neodontobutis*, *N.lani* (Zhou et al. 2022) was described recently from Longzhou Town, Guangxi, Zuojiang River basin, suggesting that more new species from these places may be uncovered.

## ﻿Comparative material

All *M.chalmersi* specimens examined for morphological comparison were deposited in the Fish Collection of the Shanghai Ocean University, China with the registration tags 76V8791, 76V8792, 76V9228, from Qiongzhong, Hainan, May 1976; HN832384 from Changjiang, Hainan, May 1983, and 070504 from Wuzhishan, Hainan, May 1983; CL2076-1 from Wuzhishan, Hainan, August 2018.

## ﻿Ethical approval

All animal procedures performed in this research were done so in accordance with the “Ethical Standards of the Shanghai Ocean University (2020)”.

## Supplementary Material

XML Treatment for Microdous amblyrhynchos

## Supplementary materials

Supplementary material 1 Pairwise distances based on COI gene among all species used in this study Data type table (excel file) https://binary.pensoft.net/file/822804This dataset is made available under the Open Database License (http://opendatacommons.org/licenses/odbl/1.0/). The Open Database License (ODbL) is a license agreement intended to allow users to freely share, modify, and use this Dataset while maintaining this same freedom for others, provided that the original source and author(s) are credited.Jiantao Hu, Chun Lan, Chenhong Li
